# Subjective Well-Being and Self-Assessed Health of Adolescents: A Longitudinal Cohort Study

**DOI:** 10.3390/ejihpe13120197

**Published:** 2023-12-04

**Authors:** Ivica Matić, Vera Musil

**Affiliations:** 1 Department of Nursing, Catholic University of Croatia, 10000 Zagreb, Croatia; 2 School of Medicine, University of Zagreb, 10000 Zagreb, Croatia; vmusil@snz.hr

**Keywords:** adolescents, subjective well-being, self-perceived health, longitudinal study, Croatia

## Abstract

Background: The aim of this study was to investigate the stability and predictors of subjective well-being and self-perceived health in adolescents over a two-year period, focusing on the importance of mental health in overall well-being. Methods: Participants in this longitudinal cohort study were surveyed at the ages of 15 (*n* = 441) and 17 (*n* = 354) through questionnaires. The data were analyzed using both descriptive and inferential statistical methods. Hierarchical regression was employed to investigate significant predictors of subjective well-being. The subjective well-being and self-perceived health dimensions showed a consistent level of stability throughout the two-year period of secondary education. Additionally, there was a significant correlation between well-being at the beginning and end of this education period. Furthermore, self-perceived health dimensions, particularly general health, vitality, and mental health, were positively associated with well-being at the end of secondary education, highlighting their role in overall subjective well-being. The regression analysis revealed that self-perceived health factors, notably “General health” and “Mental health”, significantly predicted overall subjective well-being, enhancing the model’s explanatory power beyond gender and economic status. Nevertheless, baseline subjective well-being has the strongest predictive effect on final well-being. Conclusions: This study highlights the importance of psychological and health factors, particularly mental health, that affect the overall well-being of adolescents and emphasizes the need to focus on and improve these factors in order to improve subjective well-being.

## 1. Introduction

Subjective well-being, as a person’s cognitive and affective evaluation of their life, is a central focus in psychology and health care as a multidimensional construct that combines long-term life satisfaction with short-term emotional states such as happiness. This concept emphasizes an individual’s unique perspective and recognizes that personal experiences and values shape perceptions of happiness, making it a deeply personal and subjective measure [1].

Nowadays, the importance of health for overall subjective well-being is widely recognized. Their relationship is multidimensional as numerous diseases impact health. The relationship between personal well-being and health has been extensively researched in adults [2]. Most studies focus on the impact of diseases and physical symptoms that limit functionality. Indirect effects, such as changes in work capacity, potential isolation, and increased dependence on others, have also been identified [3,4,5].

Also, some studies have been conducted on individuals suffering from various physical ailments, with pain being highlighted as the most significant variable or physical symptom associated with subjective well-being [6].

The relationship between health and personal well-being is poorly researched during adolescence because medicine perceives the young as healthy. While there has been some research on the well-being of adolescents, especially those with chronic diseases, there is a need for more specific and focused research in this area [7]. It is crucial to explore the contribution of health to the personal well-being of physically healthy young individuals, given that health itself is a multifaceted concept. Even in the absence of a diagnosed disease, an individual can experience compromised health, which in young people often pertains to the mental aspect. Therefore, it is essential to delve into various facets of health, especially mental, recognizing that health is inherently multidimensional and not a singular construct. Also, personal well-being contributes to health through the process of salutogenesis. In recent years, there has been a notable shift in adolescent health research. The traditional research approach to various risks in adolescence is directed towards a salutogenic perspective, with a direct focus on the importance of building adolescents’ strengths and capabilities, aiming to promote positive outcomes and personal well-being [8].

Adolescence, as a period of change and transition in every aspect of life, brings a multitude of potential stressors, such as shifts in responsibilities, increased academic demands, and challenges in interpersonal relationships [9,10]. While the transition through adolescence is inevitable, the pace and magnitude of changes for some adolescents may exceed their coping capacity and negatively impact their health and subjective well-being [11,12]. Most adolescents are sensitive to anything that makes them stand out, and physical appearance becomes a primary concern during these years. During adolescence, there is a risk of developing a distorted or impaired body image. This can lead to issues in various areas of functioning, from emotional and cognitive to behavioral, as this period also marks a significant development in social relationships, which primarily take place within the school context [13].

Starting high school is a notably stressful period that can impact health and personal well-being. The transition from primary to secondary school involves a significant environmental change, losing familiar peer groups and the support of primary teachers. Simultaneously, students face increased academic and social demands, requiring them to adapt to a new setting and differentiate between past behaviors and those expected in this maturing phase. The most common causes of diminished subjective well-being are linked to academic difficulties, social context, and mental issues [14]. Adolescents from disadvantaged economic backgrounds face a broader range of risks for reduced subjective well-being. They are often exposed to multiple stressors from various environments, such as a higher likelihood of physical illnesses, psychological and relational tensions within the family, and reduced social support. Additionally, a low perceived subjective well-being is observed in adolescents from large families living in impoverished areas, with unemployed parents and lower educational levels [15].

The novelty of this study lies in its exploration of the relationship between subjective well-being and self-assessed health among adolescents during secondary education. By focusing on a physically healthy adolescent population, this study fills a significant gap in existing research. Utilizing a longitudinal research design, it provides a comprehensive analysis of the stability and influencing factors of adolescent well-being over a two-year period. Longitudinal research focused on adolescents’ well-being is limited [16,17]. The research is driven by two principal questions: How stable are subjective well-being and self-perceived health among physically healthy adolescents over a two-year period in secondary education? And what are the significant predictors of subjective well-being in this group? Accordingly, we have formulated two hypotheses: (1) There will be a notable degree of stability in subjective well-being and self-perceived health among these adolescents over the two-year period; (2) mental health will emerge as a crucial predictor of personal well-being in this physically healthy adolescent cohort.

## 2. Materials and Methods

### 2.1. Participants

This was a longitudinal cohort study carried out from October 2016 to November 2018 in Zagreb, Croatia. In Croatia, secondary education is primarily conducted in grammar, vocational, and art schools, mostly located in larger cities. Zagreb, the capital and educational center of Croatia, not only hosts 15 student dormitories but also boasts 55 secondary schools. Despite different living arrangements, all students, whether living in dormitories or with their parents, attend the same schools. We included 15-year-old adolescents, selecting participants from seven high schools and six dormitories. The final group of participants included students from the chosen institutions who willingly joined the study after being fully informed about it and who met none of the exclusion criteria, such as parental objections, significant personal trauma, physical illness, or opting out of the study. At the start of the 2016/2017 academic year, the selected dormitories housed 249 first-year high school students, while the chosen schools had ten classes with a total of 260 students. The participation rate was 86% for dormitories and 89% for schools, resulting in a study group of 441 students with a mean age of 14.9 years.

Our study ensured a balanced sample in terms of gender, age, and accommodation during secondary education. Participants’ self-identified genders were recorded, and to account for potential gender-related confounders, we ensured a balanced gender representation. While the school sample typically reflected the general gender distribution, dormitories were gender-specific, prompting us to include an equal number of both male and female dormitories. Also, family structure, economic status, and place of origin were investigated. The second assessment involved 354 students, representing 80.3% of the original group. Table 1 shows the stability of the sample’s sociodemographic attributes from the start to the end of secondary education. There were no significant differences in the participants’ sociodemographic attributes between the observed periods, indicating a consistent sample.

### 2.2. Instruments

To measure the participant’s health status, the Croatian version of the SF-36 Health Status Questionnaire, licensed by the Andrija Štampar School of Public Health, was used [18]. The questionnaire comprises eight dimensions: Physical Functioning (PF), Role-Physical (RF), Bodily Pain (BP), General Health (GH), Social Functioning (SF), Role-Emotional (RE), Mental Health (MH), and Vitality (VT). For the SF-36 health status questionnaire, Cronbach’s alpha reliability coefficient yielded 0.77 in both the initial and the follow-up measurements. Personal well-being was measured using the Personal Wellbeing Index (PWI). The questionnaire consists of seven items by which the respondent assesses their satisfaction with a specific domain of subjective well-being. Satisfaction is evaluated on 11-point scales with defined endpoints of 0 (not satisfied at all) and 10 (completely satisfied). Each domain can be analyzed separately. The normative range of average group scores for Western countries is 70–90% of the maximum score. Cronbach’s α, ranging from 0.70 to 0.85, has been reported for the PWI scale [19,20,21,22]. An analysis of the scale conducted on this sample showed a reliability coefficient of 0.86 and 0.90 in the second measurement.

### 2.3. Statistical Analyses

We used the Kolmogorov–Smirnov test to assess the normality of quantitative variables. We calculated the frequencies and percentages of categorical variables. Continuous variables were presented as the mean ± standard deviation (SD). Sociodemographic differences were tested using the χ^2^-test. The independent *t*-test was used to analyze differences in subjective well-being and health at two different points in time. The Pearson correlation coefficients were calculated to determine the relationship between subjective well-being and different categories of self-perceived health. Hierarchical regression analysis was employed to determine the relationship between selected predictor variables and the criterion variable of subjective well-being. The values of *p* < 0.05 were considered statistically significant. All analyses were performed using the IBM SPSS Statistics program for Windows, version 23.0 (IBM SPSS, Armonk, NY, USA)

## 3. Results

The sociodemographic characteristics of the students remained relatively stable from the beginning to the end of secondary education. The *p*-values indicate that there were no statistically significant differences in the distributions of these characteristics over time.

Differences in the main variables related to student accommodation (parents vs. dormitories) were tested. No statistically significant difference was found in the subjective well-being with respect to student accommodation, neither at the beginning (78.26 vs. 78.63; *p* = 0.805) nor at the end of secondary education (78.65 vs. 78.90; *p* = 0.885). Additionally, no significant differences were found in the overall health perceptions of students in accommodation, either at the beginning (71.72 vs. 71.79; *p* = 0.969) or at the end of secondary education (71.45 vs. 71.24; *p* = 0.921). Table 2 shows the changes in subjective well-being and domains in self-perceived health over the course of secondary education. Notably, while the standard of living and personal relationships showed statistically significant declines by the end of this educational period, subjective well-being, personal health, achievement in life, personal safety, community connectedness, and future security displayed no significant differences. Additionally, sub-scales within the self-perceived health assessment revealed varying trends, with bodily pain registering a statistically significant decrease.

In examining the relationship between sub-scales within self-perceived health at the beginning of secondary education and subjective well-being at the end of secondary education, several patterns emerged. Subjective well-being at the beginning of secondary education showed a strong positive correlation with subjective well-being at the end of it (r = 0.425, *p* < 0.01 **), indicating that individuals who reported higher levels of subjective well-being at the outset tended to maintain those higher levels as they progressed through secondary education. Additionally, several sub-scales of self-perceived health, including general health (r = 0.285, *p* < 0.01 **), vitality (r = 0.240, *p* < 0.01 **), and mental health (r = 0.299, *p* < 0.01 **), demonstrated positive associations with subjective well-being at the end of secondary education, underlining the significant role of these facets of health in contributing to overall well-being.

Furthermore, role-physical (r = 0.198, *p* < 0.05 *) and social functioning (r = 0.187, *p* < 0.05 *) also exhibited positive correlations with subjective well-being, though somewhat weaker in magnitude. Bodily pain (r = 0.124, *p* < 0.05 *) and physical functioning (r = 0.052) displayed positive associations, but these were comparatively modest. These findings collectively underscore the importance of early well-being and certain aspects of self-perceived health as potential predictors of well-being outcomes at the end of secondary education (Table 3).

A regression analysis was conducted to identify predictors of overall subjective well-being, and the results are displayed in Table 4. These results suggest that in Model 1, gender proved to be a significant predictor with a relatively small effect on the dependent variable, while economic status did not. This model shows 2% of the variance in subjective well-being. In Model 2, after adding some domains of self-perceived health as predictor variables, gender’s effect becomes nonsignificant, while “General health” and “Mental health” emerge as significant predictors of the dependent variable. Model 2, with the additional predictor variables, has a change in R-squared (R^2^Δ) of 0.11 compared to Model 1, indicating that it explains an additional 11% of the variance in the dependent variable. This change is statistically significant (*p* < 0.001), suggesting that the additional variables improve the model’s predictive power. When baseline subjective well-being is included as a predictor, a significant effect of baseline well-being is observed (B = 0.407, β = 0.370, t = 5.565 ***). In Model 3, the impact of mental health on final well-being diminishes (B = 0.111, β = 0.119, t = 1.543), indicating that baseline well-being has a strong predictive effect on final well-being.

## 4. Discussion

The study revealed significant changes in secondary education’s impact on well-being and health. While the standard of living and personal relationships declined, other aspects remained stable. The study highlighted that initial well-being strongly predicted later well-being, as well as specific aspects of self-perceived health. Among the health variables, the dimensions of mental health and general health emerged as significant predictors, with mental health being the strongest predictor.

The average level of adolescents’ subjective well-being in this study was approximately 80% of the life satisfaction mean, and it did not significantly change during high school education. This result reflects a high level of subjective well-being among adolescents and confirms Cummins’ theory of the homeostatic model of subjective well-being, which suggests that values tend to stay within a narrow range of higher values between 70% and 80% of the life satisfaction mean. The same author, based on a systematic review of studies conducted in Western populations, found that the average subjective well-being is around 75 ± 2.5% of the life satisfaction mean [20], or 70 ± 5% of the life satisfaction mean when considering an international population [21]. The level of subjective well-being among the participants in this study is close to the upper end of the normative range, slightly above the average results reported in similar studies. Using the same measurement instrument, Tomyn et al., in two separate studies, found an average value of 75% of the life satisfaction mean [14,22] among Australian adolescents. These findings are supported by a study conducted by Casas et al. on a representative sample of 5328 adolescents from three different countries (Spain, Brazil, and Chile), where they achieved an average result of 80% of the life satisfaction mean [23]. The stability of subjective well-being concerning age is not controversial, as it aligns with the theoretical premise of homeostatic regulation of subjective well-being. Through statistical analysis of standardized results from various studies, Cummins established the average result of subjective well-being and its stability attributed to the homeostatic mechanism within each individual. While certain events may temporarily increase or decrease life satisfaction, individuals tend to return to their initial value of 75% of the life satisfaction mean over a specific period [24,25].

In this study, high average scores were obtained across all eight dimensions of health. The highest score was observed in the dimension of physical health, while the lowest average scores were found in the dimension of vitality. When comparing the results of this research to those obtained by Jureša et al., it becomes evident that adolescents, compared to the adult population, provide more favorable self-assessments of their general health, physical functioning, and social functioning and experience less pain [18]. This is expected, given that young individuals represent the healthiest segment of the general population. However, limitations due to emotional difficulties were assessed more negatively by young people, while they achieved similar results to adult participants in terms of vitality and mental health.

Establishing that there is no significant difference in health or subjective well-being between students living in dormitories and those living with their parents is crucial for challenging common stereotypes about the stress of dormitory life. This finding highlights the potential benefits of dormitory living, such as a sense of belonging, structured activities, and sports engagement, which can positively impact adolescents’ sense of coherence and well-being. It also suggests that for some students, living in a dormitory might be a less stressful or even beneficial alternative to a potentially challenging home environment [26].

Regarding various dimensions of health, a significant positive correlation between health and subjective well-being was observed, except for the association between physical functioning and well-being after two years. The strongest and statistically significant positive correlations at the beginning of secondary education, as well as after two years, were found among the variables of mental health and subjective well-being. These results align with findings in the global literature. Additionally, the correlation analysis also revealed a positive and significant association between personal well-being at the beginning and at the end of schooling, indicating that investing in strengthening subjective well-being resources today contributes to better subjective well-being in the future.

In the hierarchical regression analysis, with subjective well-being as the criterion variable and predictor variables including sociodemographic and health-related variables from the first measurement, male gender emerged as a significant predictor. The majority of previous research has confirmed that boys achieve significantly higher scores on subjective well-being compared to girls [27,28]. However, not all studies have found these differences [29]. It appears that gender may have a significant correlation, and differences in subjective well-being by gender likely stem from biological variations related to hormonal changes and psychological states [28], which could have influenced the results obtained in this study. However, after introducing health dimensions into the model, mental health and the perception of general health were identified as significant predictors. When baseline well-being is included as a predictor, a significant effect of baseline well-being is observed, indicating that baseline well-being has a strong predictive effect on final well-being. These results suggest that while mental health is an important predictor of well-being, baseline well-being has a stronger predictive effect. This does not diminish the importance of mental health but highlights the significance of baseline well-being as a key factor in predicting final well-being. It is important to emphasize that these results do not mean that mental health is not important but rather that baseline well-being may be a better predictor of final well-being in this specific context. This can be useful for understanding how well-being evolves over time and how different factors contribute to this dynamic.

The significance of this study lies in its exploration of the relationship between subjective well-being and health in adolescents, both of which are current topics related to promoting health factors. Salutogenesis remains relatively uncharted territory, primarily due to the prevailing focus on pathogenesis. Given that effective health promotion is multifaceted, it is crucial to implement promotion initiatives across various aspects of adolescent life, particularly within schools, families, and healthcare services. In our longitudinal study covering the ages of 15 to 17, it is essential to consider the significant brain maturation occurring during this critical phase of adolescence. Key developments in the prefrontal cortex, responsible for decision-making, risk assessment, and impulse control, are particularly relevant [30]. These changes can profoundly influence adolescents’ behavior and psychological profiles, affecting their ability to manage stress and adapt to new environments, such as transitioning from living at home to a dormitory. The evolution of the social brain during this period also plays a significant role in shaping how adolescents interact with their peers and authority figures [31]. These neurological developments provide a vital context for interpreting our findings, offering insights into how these biological changes intersect with and potentially influence the environmental factors explored in our study. Furthermore, the study’s strength lies in its representative sample and longitudinal design. From a practical perspective, the results of this research provide crucial information for all stakeholders within the high school system. Students and their parents can make more informed decisions regarding dormitory living based on empirical insights, while dormitory staff can derive validation for the value and justification of their work from scientifically grounded findings about the subjective well-being of their residents. For the broader community, the value lies in the realization that adolescents have satisfactory subjective well-being that should be regularly monitored, promoted, and sustained through ongoing research and the implementation of salutogenic programs within the educational system.

Nevertheless, this study faced certain limitations. Notably, all findings relied on self-reported data, which could introduce a potential bias associated with self-reporting. To mitigate this, the use of strictly anonymous responses was implemented. Additionally, the sample size was constrained due to time limitations.

Future research should delve deeper into the impact of secondary education on well-being, explore gender disparities, and assess the effectiveness of salutogenic programs in educational settings. It should also focus on diverse data sources, larger sample sizes, and longitudinal studies to enhance understanding and inform interventions and policies for adolescent well-being.

## 5. Conclusions

This study focuses on measuring the subjective well-being and self-perceived health of adolescents. It underscores the importance of mental health and general health as significant factors that contribute the most to personal well-being in adolescents. There is a clear need for further monitoring of these vital indicators during youth. Measuring personal well-being and identifying empowering factors should have a more prominent place in the scientific community and the development of interventions aimed at preserving and enhancing mental health, ultimately leading to a more satisfied youth in a time of continuous change during adolescence.

## Figures and Tables

**Table 1:** Demographic characteristics of participants at the age of 15 (*n* = 441), and 17 (*n* = 354).

Characteristic	At the Beginning of Secondary Education *n* = 441		At the End of Secondary Education *n* = 354		
	*n*	%	*n*	%	*p*
Gender					0.945
Male	197	45	159	45	
Female	244	55	195	55	
Family structure					0.594
Two parents	365	83	298	84	
Single parent	76	17	56	16	
Economic status					0.804
Lower class	14	3	14	3	
Middle class	379	86	304	86	
Upper class	48	11	36	10	
Place of origin					0.879
Urban	268	61	217	61	
Rural	173	39	137	39	
Accommodation					0.325
Parents	230	52	197	56	
Dormitory	211	48	157	44	

**Table 2:** Subjective well-being and health at two different points in time: at the beginning of secondary education and at the end of secondary education.

	At the Beginning of Secondary Education *n* = 441		At the End of Secondary Education *n* = 354		
	M	SD	M	SD	*p*
Subjective wellbeing	79.78	15.12	78.76	16.66	0.261
Standard of living	84.00	18.63	81.40	20.99	0.030
Personal health	81.10	20.35	78.70	21.48	0.060
Achieving in Life	73.50	21.37	73.90	21.50	0.747
Personal relationships	84.30	20.45	81.80	21.01	0.045
Personal safety	78.50	21.61	80.60	19.88	0.108
Community-connectedness	80.90	19.89	80.00	20.54	0.467
Future security	76.30	19.78	75.10	20.92	0.370
Self-perceived health (SF-36)					
Physical functioning	77.31	28.61	86.01	21.55	<0.001
Role-physical	74.29	30.66	75.28	31.81	0.641
Bodily pain	70.98	18.99	68.02	20.12	0.016
General health	73.15	17.91	71.36	19.16	0.077
Vitality	53.10	19.74	52.25	20.24	0.459
Social functioning	79.02	19.70	77.64	21.17	0.298
Role-emotional	64.68	35.64	64.87	40.92	0.938
Mental health	64.49	17.89	64.74	19.37	0.815

**Table 3:** Pearson correlation coefficients of study variables.

Subjective Well-Being and Self-Perceived Health Domains at the Beginning of Secondary Education	Subjective Wellbeing at the End of Secondary Education
Subjective wellbeing	0.425 **
Physical functioning	0.052
Role-physical	0.198 *
Bodily pain	0.124 *
General health	0.285 **
Vitality	0.240 **
Social functioning	0.187 **
Role-emotional	0.137 **
Mental health	0.299 **

* *p* < 0.05, ** *p* < 0.01.

**Table 4:** Hierarchical regression of subjective well-being.

		B	β	t
Model 1				
	Gender	−4.40	−0.13	−2.49 *
	Economic status	4.49	0.09	10.85
Model 2				
	Gender	−1.87	−0.56	−1.03
	Economic status	2.81	0.06	1.19
	Physical functioning	0.006	0.011	0.19
	Role-physical	0.049	0.090	10.50
	Bodily pain	−0.052	−0.059	−0.94
	General health	0.168	0.180	2.76 **
	Vitality	0.002	0.003	0.04
	Social functioning	−0.027	−0.032	−0.46
	Role-emotional	−0.013	−0.028	−0.46
	Mental health	0.207	0.223	2.86 **
Model 3				
	Gender	−2.347	−0.070	−1.351
	Economic status	1.773	0.038	0.781
	Physical functioning	−0.002	−0.004	−0.069
	Role-physical	0.040	0.073	1.275
	Bodily pain	−0.077	−0.087	−1.449
	General health	0.077	0.083	1.282
	Vitality	−0.034	−0.041	−0.597
	Social functioning	−0.052	−0.062	−0.934
	Role-emotional	−0.021	−0.046	−0.800
	Mental health	0.111	0.119	1.543
	Subjective well-being	0.407	0.370	5.565 ***

Note: Model 1, R^2^ = 0.02, *p* = 0.006; Model 2, R^2^Δ = 0.11, *p* < 0.001; Model 3, R^2^Δ = 0.18, *p* < 0.001; * *p* < 0.05, ** *p* < 0.01, *** *p* < 0.001.

## Data Availability

The data and the questionnaires from this study are available upon request from the corresponding author.
